# Subcutaneous Implantable Cardioverter-Defibrillator Lead Dislodgement Masquerading as Left Shoulder Pain

**DOI:** 10.7759/cureus.43435

**Published:** 2023-08-13

**Authors:** Yashitha Chirumamilla, Deepesh Yadav, Ghassan Bachuwa

**Affiliations:** 1 Internal Medicine, Hurley Medical Center, Flint, USA; 2 Rheumatology, University of Arkansas for Medical Sciences, Little Rock, USA

**Keywords:** lead dislodgement, atypical presentation shoulder pain, pacemaker lead displacement, transvenous pacemaker, subcutaneous implantable cardioverter

## Abstract

Implantable cardioverter-defibrillators (ICDs) have demonstrated efficacy in the prevention of sudden cardiac death secondary to cardiac arrhythmias in eligible patients. Complications with the subcutaneous ICD (S-ICD) are rarer than with the transvenous ICD but do still exist. Our patient presented four weeks after the insertion of S-ICD with complaints of left shoulder pain radiating to the chest wall and swelling over the S-ICD site. He was initially treated for rotator cuff injury and subacromial impingement syndrome but upon obtaining chest radiography was found to have a lead displacement traversing the splenic flexure of the colon. The patient was managed by a treatment team involving cardiology, surgery, and infectious disease and underwent S-ICD removal, exploratory laparotomy with splenic flexure mobilization, and completion of a four-week antibiotic course ultimately leading to reimplantation of S-ICD.

## Introduction

Sudden cardiac death associated with cardiac arrhythmias such as sustained ventricular tachycardia or ventricular fibrillation is one of the leading causes of cardiovascular mortality. Implantable cardioverter-defibrillators (ICDs) have been evaluated in several large-scale clinical trials and shown to reduce mortality in eligible populations [1]. Since the introduction of subcutaneous ICDs (S-ICDs), they have become the standard for primary and secondary prevention of sudden cardiac death, but complications do occur. One of the rare complications is lead displacement. Our patient was found to have the same but initially presented with complaints of left shoulder pain, a symptom previously not documented to be associated with lead displacement after S-ICD implantation.

## Case presentation

A middle-aged male with a past medical history of ischemic cardiomyopathy status post S-ICD implantation four weeks earlier, essential hypertension, obstructive sleep apnea, and class II obesity presented to the emergency department with complaints of worsening left shoulder pain, now radiating to the left anterior chest wall and swelling over his S-ICD site. His shoulder pain had been persistent for the last four weeks and resulted in several visits to his cardiologist and primary care physician (PCP) for further evaluation. He reported that two days earlier, he rolled over in his sleep and since then has had swelling over the S-ICD site. He denied fevers but did complain of intermittent diaphoresis and chills. Upon arrival, his vitals were significant for tachycardia with a pulse of 129 beats per minute but otherwise hemodynamically stable and afebrile. On physical examination, he had diffuse tenderness of the entire left shoulder. Active range of motion was associated with pain; however, passive range of motion was intact with no crepitance or overlying skin changes. At the left lateral chest wall, the site of S-ICD insertion, there was warmth and tenderness along with fluctuance. The wound was approximated with no discharge or bleeding.

**Figure 1:**
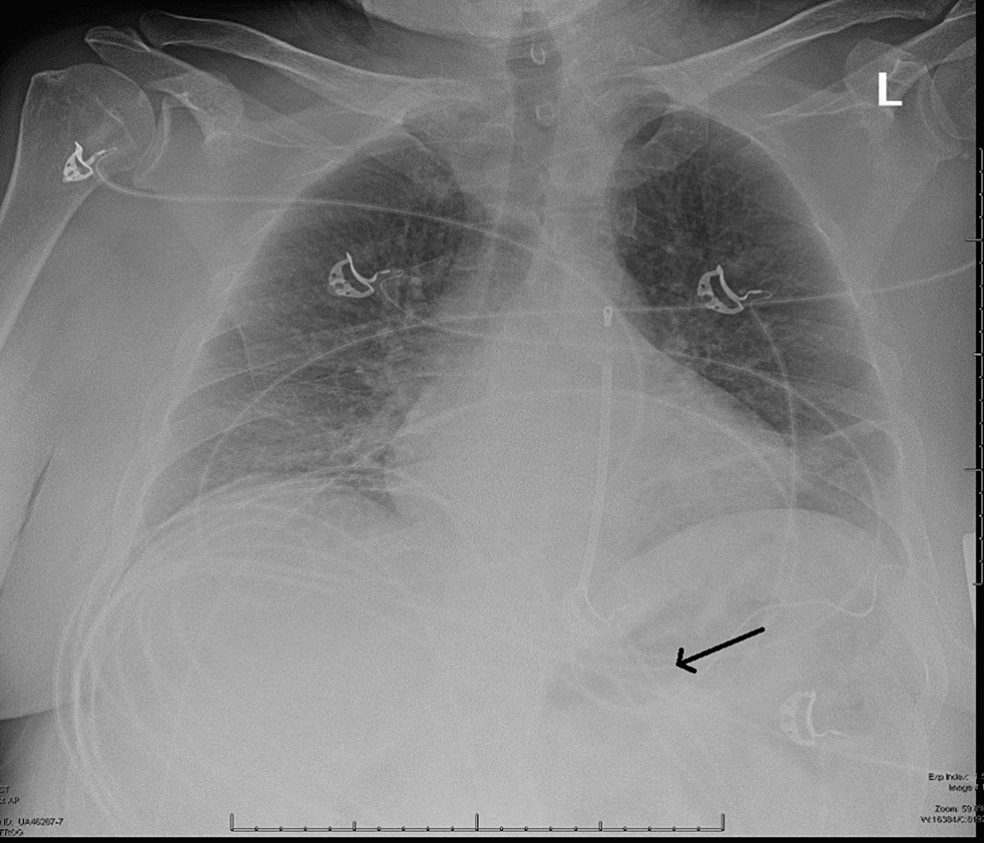
Initial chest radiography on presentation showing mild pulmonary edema and displaced S-ICD lead (black arrow). S-ICD: subcutaneous implantable cardioverter-defibrillator

An electrocardiogram (EKG) done revealed sinus tachycardia with no signs of acute ischemia. Cardiac enzymes were also negative for acute myocardial infarction. Other laboratory markers were relatively normal. The patient was initially evaluated with a single-view chest radiography, which identified mild pulmonary edema and a device on the left chest but failed to properly identify the S-ICD and its leads (Figure 1).

**Figure 2:**
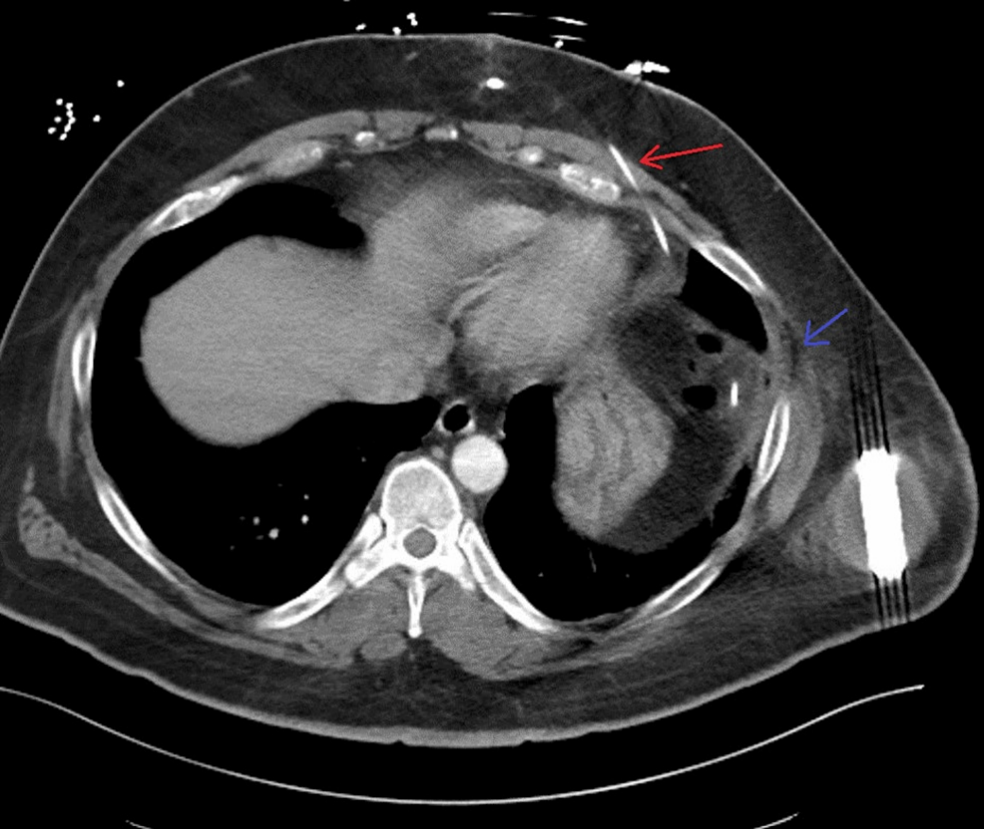
Computed tomography demonstrating the intra-abdominal approach of S-ICD lead traversing the splenic flexure of the colon with the tip (red arrow) located in the anterior subcutaneous tissues of the chest wall and a focus of gas with pleural thickening (blue arrow). S-ICD: subcutaneous implantable cardioverter-defibrillator

Given the patient’s recent procedure and new-onset swelling, computed tomography (CT) of the chest was obtained. CT of the chest revealed the S-ICD lead traversing between the left seventh and eighth rib with soft tissue swelling of the overlying chest wall and small foci of gas around the lead wire. A portion of the S-ICD lead traversed intra-abdominally into the splenic flexure of the colon and proceeded anterosuperiorly between the left sixth and seventh rib costochondral junction into the subcutaneous tissues of the chest just to the left of the midline. There was also focal pleural thickening and a focus of gas adjacent to the left S-ICD lead site (Figure 2).

The patient started having complaints of achy left shoulder pain two days after the insertion of his S-ICD and was evaluated by his PCP. He had diffuse pain in the left shoulder, extending to the neck, exacerbated by overhead movements of the left arm and lying on the left shoulder. He was initially suspected to have rotator cuff tendinopathy and underwent a left shoulder X-ray, which was negative for acute bony abnormalities. He was prescribed oral pain medications and advised to apply ice. The patient also followed up with his cardiologist; his S-ICD insertion site was healing well and elicited no tenderness. He was advised to continue current management regarding his left shoulder pain. However, his pain persisted despite oral pain medication use. At his follow-up visit with his PCP, he was diagnosed with subacromial impingement syndrome substantiated by his pain with overhead movements and lying on the left shoulder along with diffuse tenderness of the acromioclavicular joint with normal strength. He was treated with an intramuscular glucocorticoid injection to the shoulder bursa. The patient never had relief from the pain, and it ultimately prompted him to visit the emergency department.

**Figure 3:**
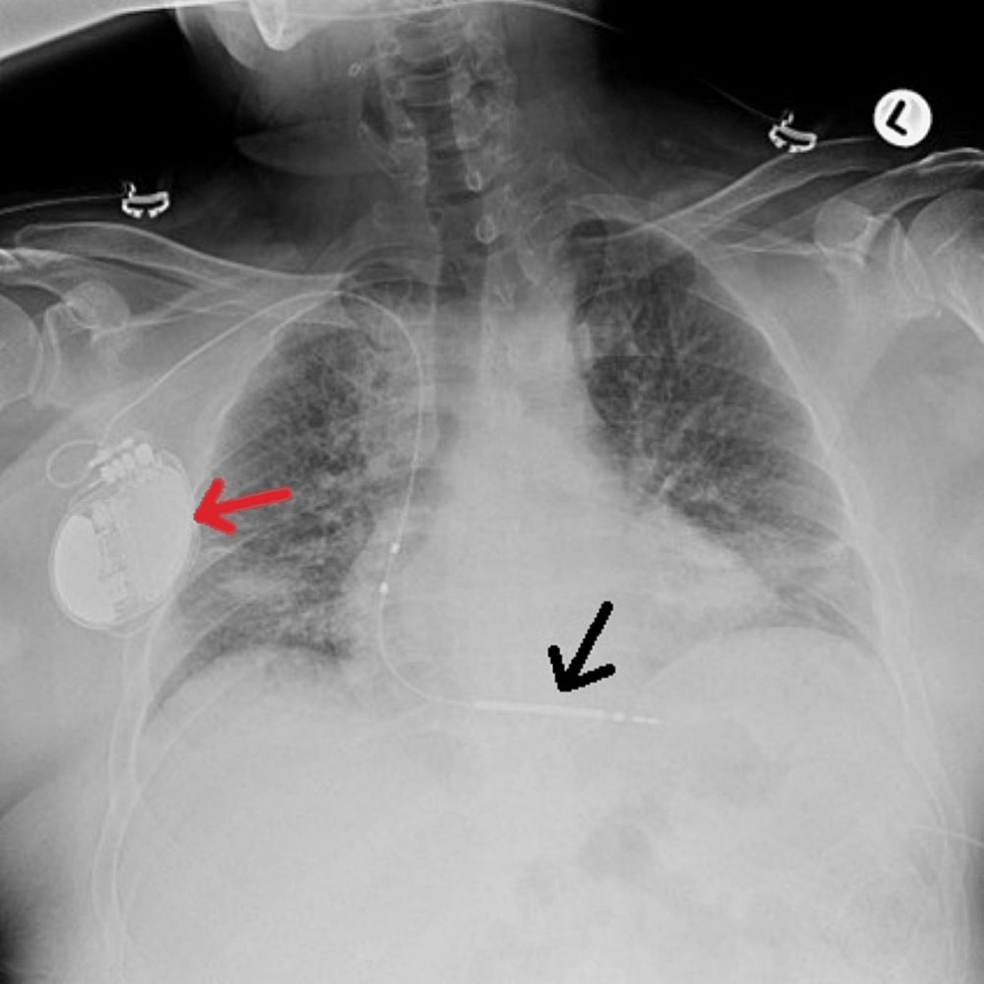
Chest radiography obtained after reimplantation of the S-ICD (red arrow) in which pneumothorax is ruled out and the lead placement is optimal with the tip (black arrow) terminating near the ventricular apex. S-ICD: subcutaneous implantable cardioverter-defibrillator

The patient was promptly started on broad-spectrum antibiotics given the foci of gas visualized on the CT imaging of the chest. Cardiology, surgery, and infectious disease were consulted, and a collective decision was made for the patient to undergo S-ICD removal and exploratory laparotomy with mobilization of the splenic flexure and segmental colectomy. During the procedure, it was confirmed that the lead entered into the left lateral upper quadrant, traversed through the splenic flexure of the colon, exited the splenic flexure, and finally traversed back through the anterior abdominal wall. Blood cultures remained negative; however, both tissue cultures and removed foreign body cultures were positive for the growth of methicillin-resistant *Staphylococcus aureus* (MRSA). Antibiotics were changed according to the culture sensitivities, and the patient was treated with daptomycin for a total of 14 days. Due to his chest wall incision and dressing, he could not be properly fitted for a LifeVest and remained admitted until an S-ICD implantation could be performed once again as he was at high risk for cardiac arrhythmias and sudden cardiac death. Once cleared by infectious disease specialists, he underwent a successful single-chamber S-ICD insertion in the right upper chest (Figure 3).

Following repeat S-ICD insertion, the patient was discharged home with seven additional days of antibiotics. He followed up with his PCP and reported a complete resolution of his shoulder pain. He was seen at the infectious disease clinic after his course of antibiotics and found to have no signs of ongoing infection. He followed up with cardiology and had no further issues with his S-ICD.

## Discussion

Currently, the implantable device options available include transvenous ICD and more recently S-ICD. S-ICDs are being favored as they have been shown to reduce the immediate complications associated with transvenous ICDs such as pneumothorax and cardiac perforation and long-term complications such as systemic infections [2]. Our patient did develop a localized infection but never went on to develop bacteremia or other systemic infections. The EFFORTLESS S-ICD was an observational study conducted to evaluate the long-term efficacy of S-ICD and patients’ quality of life in 50 centers that had approved the use [3]. A report on the three-year follow-up of the full EFFORTLESS cohort demonstrated that the 30-day and 360-day complication rate with S-ICD implantation was 0.3% and 2%, respectively. The most frequent complications were inappropriate shocks, discomfort, and infections [4].

Dislodgement of the leads can be “micro” or “macro.” Micro-dislodgements are not radiographically evident and involve a minimal displacement of the lead tip [5]. Lead macro-dislodgement (LMD) is defined as the gross displacement of the lead of an implantable cardiac electronic device that is identified using chest radiography or other imaging modalities. It is a very rare complication of transvenous ICDs; a population-based study with a cohort of 1,074 described the phenomenon in 1%-8% of patients [6]. Several names have been assigned to describe different variants of LMD, including twiddler and reel most commonly but also reverse twiddler, reverse reel, and ratchet. The possible etiologies for LMD are device manipulation, mechanical forces either during and after the procedure, the creation of a large pocket for the generator, and redundant subcutaneous tissue [7].

Although LMD is more common with transvenous ICDs, there are a few reports of the complication with S-ICDs as well. A 16-year-old patient had presented due to inappropriate shocks from his S-ICD and was found to have a lead displacement secondary to device manipulation, a form of twiddler’s syndrome. Some of the risk factors associated with twiddler’s syndrome are female gender, obesity, pediatric or elderly population, psychiatric illness history, and device-pocket size mismatch [8]. Another case report demonstrated LMD in a 16-year-old patient with S-ICD found incidentally on chest radiography, which was suspected to be secondary to strenuous exercise shortly after the procedure [9]. Our patient also presented with worsening pain and new-onset swelling after movement in his sleep, which could have caused some mechanical force and manipulation of the device. There are two case reports of a 69-year-old female and an eight-year-old male, both with transvenous ICD lead displacement and a presentation of intractable hiccups due to irritation of the phrenic nerve [10,11]. Our patient also likely had irritation to the phrenic nerve causing referred pain to the left shoulder to be his primary complaint. This particular presentation appears to be unique in regard to S-ICD LMD.

## Conclusions

Clinicians should be cautious of patients post-ICD placement presenting with new-onset symptoms that are seemingly unrelated, such as shoulder pain or hiccups. A higher clinical suspicion should be maintained for LMD, particularly in patients with multiple risk factors such as female gender, obesity, pediatric or elderly age group, and psychiatric illness history. Even patients lacking the typical risk factors should be extensively counseled regarding the avoidance of physical exertion and device manipulation after ICD implantation.
